# Caring for nursing home residents with COVID-19: a “hospital-at-nursing home” intermediate care intervention

**DOI:** 10.1007/s40520-021-01959-z

**Published:** 2021-08-20

**Authors:** Enrico Benvenuti, Giulia Rivasi, Matteo Bulgaresi, Riccardo Barucci, Chiara Lorini, Daniela Balzi, Antonio Faraone, Giacomo Fortini, Gabriele Vaccaro, Ilaria Del Lungo, Salvatore Gangemi, Sante Giardini, Cecilia Piga, Eleonora Barghini, Serena Boni, Giulia Bulli, Paolo Carrai, Andrea Crociani, Aldo Lo Forte, Letizia Martella, Simone Pupo, Irene Marozzi, Giulia Bandini, Primo Buscemi, Claudia Cosma, Lorenzo Stacchini, Lorenzo Baggiani, Andrea Ungar, Enrico Mossello, Guglielmo Bonaccorsi, Giancarlo Landini

**Affiliations:** 1 Geriatric Unit, Santa Maria Annunziata Hospital, Local Health Unit “Toscana Centro”, Florence, Italy; 2 Division of Geriatric and Intensive Care Medicine, Careggi Hospital, University of Florence, Azienda Ospedaliero-Universitaria Careggi, Largo Brambilla 3, 50139 Florence, Italy; 3 Department of Health Science, University of Florence, Florence, Italy; 4 Epidemiology Unit, Local Health Unit “Toscana Centro”, Florence, Italy; 5 Department of Internal Medicine, San Giovanni di Dio Hospital, Florence, Italy; 6 Division of Internal Medicine, Careggi Hospital, Florence, Italy; 7 Department of Territorial Health Network, Florence, Italy; 8 Department of Internal Medicine, Santa Maria Nuova Hospital, Local Health Unit “Toscana Centro”, Florence, Italy

**Keywords:** Nursing home, Mortality, COVID-19, Intermediate care, Integrated medicine, Hospital-at-home

## Abstract

**Background:**

Nursing home (NH) residents have been dramatically affected by COVID-19, with extremely high rates of hospitalization and mortality.

**Aims:**

To describe the features and impact of an assistance model involving an intermediate care mobile medical specialist team (GIROT, Gruppo Intervento Rapido Ospedale Territorio) aimed at delivering “hospital-at-nursing home” care to NH residents with COVID-19 in Florence, Italy.

**Methods:**

The GIROT activity was set-up during the first wave of the pandemic (W1, March–April 2020) and became a structured healthcare model during the second (W2, October 2020–January 2021). The activity involved (1) infection transmission control among NHs residents and staff, (2) comprehensive geriatric assessment including prognostication and geriatric syndromes management, (3) on-site diagnostic assessment and protocol-based treatment of COVID-19, (4) supply of nursing personnel to understaffed NHs. To estimate the impact of the GIROT intervention, we reported hospitalization and infection lethality rates recorded in SARS-CoV-2-positive NH residents during W1 and W2.

**Results:**

The GIROT activity involved 21 NHs (1159 residents) and 43 NHs (2448 residents) during W1 and W2, respectively. The percentage of infected residents was higher in W2 than in W1 (64.5% vs. 38.8%), while both hospitalization and lethality rates significantly decreased in W2 compared to W1 (10.1% vs 58.2% and 23.4% vs 31.1%, respectively).

**Discussion:**

Potentiating on-site care in the NHs paralleled a decrease of hospital admissions with no increase of lethality.

**Conclusions:**

An innovative “hospital-at-nursing home” patient-centred care model based on comprehensive geriatric assessment may provide a valuable contribution in fighting COVID-19 in NH residents.

**Supplementary Information:**

The online version contains supplementary material available at 10.1007/s40520-021-01959-z.

## Introduction

Since the first outbreak of coronavirus disease 2019 (COVID-19), nursing homes (NHs) have become epicenters for transmission of the Severe Acute Respiratory Syndrome Coronavirus 2 (SARS-CoV-2) and NH residents have shown disproportionately high risk of contracting the infection [1–4]. Older adults living in NHs are a highly vulnerable population, as it includes frail individuals with high rates of multimorbidity, frailty, disability and cognitive decline, which determine extreme clinical complexity and an increased risk of poor outcome of acute illnesses [5, 6]. As expected based on these premises, SARS-CoV-2 infection had significantly higher lethality among NH residents in comparison with the general population, even when asymptomatic [7]. According to available data, COVID-19 lethality rates in the NH setting exceed 45% [1, 8], whereas 2–12% was estimated for the general population in different countries and in different moments of the pandemic [9–11]. Moreover, all-cause mortality in NHs showed a twofold increase as compared to previous years [3].

In addition, significant hospitalization rates have been reported in SARS-CoV-2-positive NH residents [7, 12, 13], substantially contributing to hospital overcrowding. In NH residents, hospital admission frequently results in prolonged stays and hospital-related complications such as delirium, infections, malnutrition and functional decline, that negatively impact patients’ prognosis [14–16]. These complications have been magnified during the COVID-19 pandemic, due to patients’ distress deriving from isolation, loneliness, and staff use of personal protective equipment (PPE) with consequent sensory deprivation [17–19]. Moreover, in the hospital setting, frailer residents may experience overtreatment, i.e. receive unnecessary burdensome treatments which provide greater harms than benefits [16, 20]. Therefore, hospitalization of NH residents may lead to considerable healthcare system burden and increased public health costs, with limited or no improvement in patients’ prognosis and quality of life.

Available literature data indicate that NH COVID-19-related mortality has reduced during the second wave, possibly due to improved knowledge of disease management and transmission control strategies [21]. Moreover, implementing on-site care in NHs based on a comprehensive geriatric assessment [22, 23] may help to reduce unnecessary hospital admission, complications and costs, while providing adequate patient care tailored to individual frailty and functional level.

Prior to the pandemic, approximately 3500 individuals (mean age 85 years) lived in 126 NHs in Florence Health District in Tuscany, Italy. Although included in the healthcare service network, NHs received no direct control from hospitals, and residents’ health issues were usually managed by general practitioners (GPs), with direct access to hospital care in the case of severe acute illness. Additionally, dedicated medical care was poorly represented in the studied NHs.

In response to the high number of COVID-19 outbreaks in the NH setting and the consequent considerable hospitalization rates, at the beginning of April 2020, the Tuscany Region mandated that hospitals set up specialized multidisciplinary clinical and logistic teams to provide support to GPs and NH staff, organizing Intermediate Care (IC) assistance on the model of transitional care [24, 25], to be provided on-site at the NHs. A “hospital-at-nursing home” mobile multidisciplinary team (GIROT, Gruppo Intervento Rapido Ospedale Territorio), including geriatricians and internal medicine specialists and nurses, was created to deliver urgent care to NH residents with COVID-19 directly at their bedside, thus introducing an innovative organizational model for outbreak management.

The present paper describes the GIROT activity, that was set up in Florence during the first wave of the pandemic and reached its full potential during the second wave. Moreover, we report the rate of hospitalization for COVID-19 during the first and the second wave, contemporarily taking into account infection lethality.

## Methods

In April 2020, the Tuscany Region created a local multidisciplinary Task Force for disease management in the NHs and adopted a resolution that allowed hospital specialists to be in charge of SARS-CoV-2-positive residents (Regional Decision no. 28, April 7th 2020). The GIROT was thus set up to provide residents’ care inside facilities with active COVID-19 outbreaks, in collaboration with GPs and non-specialist medical doctors from Continuity Care Teams (CCTs, in Italian “Unità Speciali di Continuità Assistenziale, USCA”). In particular, the GIROT activity started during the first wave of the pandemic (March–April 2020) as an emergency response to extremely high rates of infection, hospitalization and mortality observed in the NHs of Florence Health District since the beginning of the pandemic. This preliminary experience of on-site geriatric care was then optimized through a “learning from experience” process, including the standardization of operative protocols for disease treatment and transmission control, which led to the creation of a structured healthcare and organizational model that was entirely implemented during the second wave of the pandemic (October 2020–January 2021). The GIROT intervention was developed on the model of a Canadian hospital-NH partnership involving specialized teams from hospital, that provided a multi-phase emergency response to a 126-bed NH experiencing a COVID-19 outbreak [24]. The team emergency response in the NH setting included additional staffing, infection prevention and control strategies, occupational health and operational support [24]. A similar hospital-community partnership had been developed before the pandemic in Northern Italy, where hospital-based multidisciplinary mobile unit teams delivered specialist care directly to the NHs with the final purpose to reduce hospital admissions [25].

### General coordination and transmission control

Members of the GIROT included geriatricians and internal medicine specialists, nurses, and other healthcare professionals, as detailed in Table 1. The aim of the GIROT was to set up IC units on-site in the NHs, with nursing care guaranteed 24 h and specialist medical care available on a daily basis for a minimum of 6 h a day. The GIROT activities involved the following tasks (Table 2):

- Prompt environmental interventions to limit transmission, including provision of PPE and setup of “COVID-19 bubbles” to isolate infected residents;
- Setting up of systematic residents’ and staff testing for SARS-CoV-2 infection, including infection screening with viral swabs every 2 weeks;
- Supply of nurses and other healthcare workers in case of staffing shortage;
- Direct provision of oxygen, first-line medications (Supplementary Table 1), and caloric nutritional supports;
- Activation of palliative care services, when deemed appropriate;
- Discontinuation of isolation at the end of the infection, according to a symptom-based approach [26].

**Table 1:** GIROT members and roles

Member	Role
Medical specialists ∙ Geriatricians ∙ Internal medicine specialists	Team coordination and direction Clinical management of COVID-19 infection (diagnostic exams, clinical evaluation and therapy) Prevention and management of geriatric syndromes Communication with families End of patients’ isolation after infection
Local Health District Nurse	Advice and support to NH nurse management Staff training for COVID-19 on PPE use and cleaning procedures Setting up of residents’ and staff testing Advanced nursing care, including management of complicated pressure sores and feeding tubes
Local Health District Direction	Nursing care coordination and direction Staffing management, including supply of health workers in case of staffing shortage Provision of PPE stocks
Local Health District Physiotherapy	Conventional geriatric rehabilitation Respiratory training Coordination of patients’ mobilization
Palliative specialists	Early palliative care Provision of palliative medications Communication with families
Public hygiene experts and occupational health professionals	Setup of COVID-19 “bubbles” and dedicated pathways including donning and doffing stations Other environmental interventions for transmission control, including creation of COVID-19 signs and posters Management of NH staff occupational health issues End of patients’ isolation after infection

*PPE* personal protective equipment

**Table 2:** GIROT interventions provided in each nursing home during the outbreak

*At the beginning of the outbreak*
Environmental interventions to limit transmission: cleaning procedures, room changes and setup of “COVID-19 bubbles” including with donning and doffing stations
Residents’ and staff testing to identify all SARS-CoV-2 cases
Use of ID bracelets to favor residents’ identification (particularly in presence of external staff)
Comprehensive geriatric assessment of SARS-CoV-2-positive residents and risk stratification according to symptoms severity (Green/Yellow/Red code); medical therapy review and optimization (including COVID-19 protocol-based therapy), blood testing
Direct provision of oxygen and first-line medications (Table 2) within 24 h of outbreak
Supply of caloric nutritional supports including specific diets for dysphagia
Identification of staff shortage and supply of nurses and other healthcare workers from local hospitals as appropriate
Provision of PPE and staff training on appropriate PPE use and other transmission control procedures
*During the course of the outbreak*
Regular medical assessment according to color coding, including interventions for prevention and management of geriatric syndromes
Activation of palliative comfort-based care services, when deemed appropriate based on comprehensive geriatric assessment
Regular residents’ and staff SARS-CoV-2 testing for infection monitoring
Daily clinical report to GPs and regular update to families
Discontinuation of residents’ isolation at the end of the infection, according to a symptom-based approach^a^

*PPE* personal protective equipment

^a^The symptom-based approach [26] allowed patients to be released from isolation also in presence of a positive virus test, provided that they were asymptomatic for at least 21 days

The GIROT provided first-line diagnostic assessment, including electrocardiogram, hemogasanalysis, bedside chest ultrasound and portable radiological supports, as deemed necessary. On-site blood tests were performed in all SARS-CoV-2-positive residents. Daily briefings occurred to organize the activity.

The GIROT activity was developed in concert with NHs staff, including nurses, social and health professionals and physiotherapists. All of them received training by the GIROT professionals in the use of PPE and in hygiene and transmission control measures. Moreover, some of them constituted multidisciplinary teams caring for SARS-CoV-2-positive residents inside the “COVID-19 bubbles”, thus allowing to provide IC assistance in a “familiar” atmosphere. GPs were regularly informed about residents’ health status and participated in clinical decisions. Finally, in ten amongst the involved NHs, the GIROT professionals were also supported by local physicians operating on-site in the facility.

### Protocols of geriatric management

Each resident underwent risk stratification according to COVID-19 symptoms severity and a comprehensive geriatric assessment. Risk stratification was carried out using the following color code:

- Red code: respiratory failure, severe gastrointestinal symptoms, delirium, hypo-/anorexia.
- Yellow code: mild symptoms such as fever, cough, flu-like symptoms.
- Green code: no or minimal symptoms.

Patients identified with a red code received daily medical assessment from the GIROT. Those identified with a yellow code received daily medical assessment from the CCTs and additional specialist assessment from the GIROT at least twice a week. The CCTs were in charge of patients identified with a green code, although specialist care could be required if deemed appropriate.

The GIROT pursued a goal of care conversation with GPs, NH staffs and families to understand the premorbid clinical and functional status of each resident and his/her treatment preferences, with the final purpose to define treatment goals (i.e., active medical management or palliative care) as well as the preferred place of care (i.e., hospital admission or on-site NH management). To avoid unnecessary hospital admissions, on-site management was preferred in presence of at least two among the following conditions: very low functional autonomy (< 3 basic activities of daily living preserved, among bathing, dressing, toileting, transferring, continence and eating), severe dementia (stage 7 of Global Deterioration Scale) [27], severe impairment of walking capacity (need of intensive walking assistance or in wheelchair/bedridden). Direct admission to acute care wards or in-hospital IC was, however, discussed for any resident with severe symptoms, i.e. red code.

On-site medical management consisted of a protocol-based treatment strategy also including intravenous drug administration and hydration, and low-flow supplemental oxygen when necessary. The treatment protocol (Supplementary Table 1) was based on available evidence [28–32] and approved by a consensus of local specialists including geriatricians, infectious disease and internal medicine specialists. Medical management also included prevention and management of the major geriatric syndromes, such as delirium, hypokinetic syndrome, pressure sores, urinary incontinence, constipation and malnutrition (Supplementary Table 2).

Residents’ families received up-to-date clinical information by the NH staff and by the GIROT medical members at least twice a week.

The present paper reports on aggregate administrative data recorded by the Healthcare District from an outbreak intervention undertaken as part of public health practice for the purpose of controlling COVID-19 disease transmission. As such, the work did not require Research Ethics Committee approval.

Recorded data included:

- Age and gender of the residents on March 1st, for the NHs involved in the first wave, and on October 1st, 2020, for the NHs involved in the second wave;
- Total number of residents in each NH at the beginning of the outbreak and number of cases with positive SARS-CoV-2 testing;
- Rate of hospital admission and death during COVID-19 outbreak among residents testing positive.

### Statistical analysis

Chi2 test was used to compare the rate of positive SARS-CoV-2 testing, hospitalization and lethality between the two waves, considering an alpha level of 0.05 as statistically significant. For each participant, hospitalization and death data were censored at the time of the end of the outbreak in the hosting NH. Deaths after hospital admission were included in the analysis.

## Results

The number of NHs which were struck by SARS-CoV-2 infection and hosted GIROT activity was 21 (1159 residents) and 43 (2448 residents) in the first and second wave, respectively (Table 3). Their capacity ranged from 30 to 160 beds, with an average of 55 and 57 beds per facility in the first and second wave, respectively. Among 43 NH facilities involved in the second wave, only one had experienced an outbreak during the first wave. The percentage of SARS-CoV-2-positive residents was significantly higher in the second wave in comparison with the first one (64.5% vs. 38.8%), while hospitalization rate among SARS-CoV-2-positive residents was significantly higher in the first wave than in the second one (58.2% vs 10.1%). Lethality of SARS-CoV-2 infection was also higher in the first wave as compared to the second (31.1% vs 23.4%) (Table 3).

**Table 3:** Hospitalization and mortality rates in nursing home residents receiving care by the GIROT: comparison between first and second wave of the pandemic

	First wave March 1st 2020–April 30th 2020	Second wave October 1st 2020–January 31st 2021
Number of nursing homes	21	43
Number of residents	1159	2448
Mean age (years)	83.9	82.8
Female residents	830 (71.6%)	1728 (70.6%)
Number of COVID-19-positive residents	450	1578
% of COVID-19-positive residents*	38.8%	64.5%
Number of deaths	140	369
Number of residents admitted to hospital	262	160
Hospitalization rate among COVID-19-positive residents^†^	58.2%	10.1%
Lethality rate among COVID-19-positive residents ^‡^	31.1%	23.4%

^*^Chi2 test (1df): 210.0; *p* < 0.001

^†^Chi2 test (1df): 491.2; *p* < 0.001

^‡^Chi2 test (1df): 11.1; *p* < 0.001

## Discussion

The present study describes an innovative model of integrated medicine which was developed in the NH setting during the COVID-19 pandemic and was completely structured during the second wave. In addition, we report rates of hospitalization and lethality in SARS-CoV-2-positive residents during the first and second wave of the pandemic, showing a significant increase in the rate of infection but a significant decrease of hospitalization during the epidemic course. Lethality was also significantly lower during the second wave.

The huge drop of the hospitalization rate between the first and the second wave of the pandemic paralleled the implementation and optimization of the GIROT activity. Indeed, during the first wave, more than half of SARS-CoV-2-positive residents were admitted to acute and intermediate care hospitals, while only one in ten was hospitalized during the second wave of the pandemic. The hospitalization rate observed during the second wave was much lower than that described by previous studies, reporting that about 20% of SARS-CoV-2-positive NH residents have been admitted to hospital during COVID-19 disease, with rates up to 45% among severely symptomatic cases, both in large US studies as well as in Italian samples [7, 13, 33]. The results we observed can be explained by the progressive upgrading of COVID-19 “bubbles” to create on-site IC units, where SARS-CoV-2 infected patients could be isolated from the negative ones while receiving appropriate multidisciplinary care, thus avoiding the need for hospitalization. The increased rate of SARS-CoV-2-positive residents during the second wave is parallel with the increased rate of infected NH healthcare workers that was observed in Florence Health District (data not shown). This trend is consistent with the more widespread infection that was reported in the general population during the second wave in comparison with the first one, possibly resulting in multifocal infection outbreaks inside the NHs struck by the pandemic.

In our sample, we also observed a 31% lethality rate among NH residents testing SARS-CoV-2 positive during the first wave, thus confirming the vulnerability of this population, in agreement with existing scarce literature data. In a study by Tang et al., 30-day cumulative mortality in SARS-CoV-2-positive NH residents ranged from 14% in asymptomatic individuals to 39% in those with 2 or more signs or symptoms, as compared to 7% mortality rate in SARS-CoV-2-negative residents [7]. Consistent data are reported by McMichael et al. (34%) and Arons et al. (26%) in skilled nursing facilities [12, 34]. In Dutch NHs, almost half of SARS-CoV-2-positive residents died within 30 days and mortality rate was three times higher than in negative residents [2, 8]. In the UK, 907 cases of SARS-CoV-2 infection were recorded in 189 care homes leading to a 48% lethality rate [1]. In the present study, infection lethality was significantly lower during the second wave of the pandemic, reaching 23.4% (*p* < 0.001), similar to what was observed in a sample of Italian NHs in Veneto region and in a large sample of US NHs [13, 33]. The fall in NH COVID-19 deaths reported in our study is consistent with a recent study by Ioannidis et al., describing a significant reduction in NH mortality rates in the second vs the first wave in 8 countries [21]. This phenomenon is likely attributable to different contributing factors, as the authors suggest. First, a “learning from experience” process has taken place during the pandemic, leading to the development and step-by-step improvement of COVID-19 treatment protocols and of strategies for infection control, e.g. cleaning procedures, patients’ isolation and testing, that have become routine during the second wave. Moreover, the impressive lethality rates observed during the first wave have prompted interventions aimed to improve NH residents’ care during the COVID-19 outbreak, including the “hospital-at-nursing home” model described in the present paper. It should be considered that, in some countries, NH COVID-19 mortality did not vary or even increased in the second vs the first wave of the pandemic [21], probably due to pre-existent weaknesses and inefficiencies in the local care systems [35, 36].

This supports the hypothesis that active intervention strategies aimed to potentiate patients’ care in the NH setting may contribute to explain why the marked decrease of hospitalizations was not coupled with any increase of mortality. Indeed, a “hospital-at-nursing home” environment might have allowed the implementation of good geriatric practice and on-site provision of effective treatments for COVID-19, thus avoiding hospital admission and hospital-related complications that more frequently occur in older patients, e.g. nosocomial infections, immobilization, functional decline, isolation and loneliness. In fact, it is widely recognized that COVID-19 negative impact on older adults’ health status is at least partly related to separation from their life environment [18]. This affection deprivation is further exacerbated in the hospital setting, while it may be attenuated in the NHs thanks to staff members creating a more confident and familiar atmosphere [17]. In addition, although we are unable to provide numerical data, we perceived that residents’ families appreciated this hospital-at-nursing home taking care approach.

### Limitations

The above data should be interpreted in the context of some limitations. First, we present the data of a multifactorial intervention, and we are unable to assess which component has played a major role in hospital admission decrease. Moreover, we cannot exclude that some differences between NH residents testing SARS-CoV-2 positive in the two waves of the pandemic may have influenced patients’ outcomes. However, criteria for NH admission are standardized across the facilities involved in the study, based on a comprehensive multidisciplinary assessment carried out by the dedicated team of the Local Health Unit. It can also be argued that less severe or asymptomatic cases might have been missed during the first wave, as reported in community-dwelling Italian subjects at the beginning of the pandemic [37]. Yet, from the beginning of the pandemic period, all NH residents of Florence Health District received a viral swab every 2 weeks for the purpose of early identification and control of COVID-19 outbreaks. Therefore, we pose that infection under-diagnosis is unlikely in the present population. A cohort effect has been hypothesized in previous studies and may potentially contribute to explain the reduction in residents’ lethality during the second wave. Indeed, frailer residents may have died during the first wave, while a large proportion of other residents has been infected and immunized. Yet, this cohort effect is unlikely to have influenced the results of our study, as distinct NH facilities were involved in the two waves of the pandemic.

We may thus suppose that the change of healthcare organization described in the present paper may help to explain the marked decrease of hospitalization rate. In addition, the lower lethality rate observed in the second wave may have further contributed to reduce hospital admissions. In the present study, mortality data were censored at the end of each NH outbreak, which precluded the analysis of COVID-19-related long-term deaths.

This paper presents a real life experience which is evolving, and data collection is still in progress. Future studies will provide additional details on clinical and functional features of involved NH residents allowing to better investigate predictors of adverse outcomes including mortality.

## Conclusions

Nursing home residents were dramatically overwhelmed by COVID-19 epidemic, with impressively high mortality and hospitalization rates. An innovative on-site patient-centred care model based on comprehensive geriatric assessment may provide a valuable contribution in fighting COVID-19 outbreak in this vulnerable population, with a globally lower health care burden.

## Supplementary Information

Below is the link to the electronic supplementary material.

Supplementary file1 (PDF 124 KB)

## Data Availability

The dataset analyzed during the current study is available from the corresponding author on reasonable request.
