# Supplementary material for: Caring for nursing home residents with COVID-19: a “hospital-at-nursing home” intermediate care intervention
Source: Aging Clin Exp Res. 2021 Aug 20;33(10):2917–24. doi: 10.1007/s40520-021-01959-z (PMC8378521; doi:10.1007/s40520-021-01959-z)
Supplement: Supplementary file 1 — Supplementary file1 (PDF 124 KB) [file 40520_2021_1959_MOESM1_ESM.pdf]

Table S1. GIROT treatment protocol for COVID-19 infection.

Patient colour code	Therapy	Comments
Green code (asymptomatic patients)	Prophylactic enoxaparin (4.000 U.I. aXa/0,4 ml - dose adjustment in case of severe renal impairment)	Administered for at least 14 days given the high prevalence of reduced mobility
Yellow code (mild to moderate symptoms)	Prophylactic enoxaparin (4.000 U.I. aXa/0,4 ml - dose adjustment in case of severe renal impairment)<br><br>Dexamethasone (6 mg daily im) or oral prednisone (25 mg daily) in patients with at least moderate symptoms, e.g. cough, fever lasting 3 days or more<br><br>Lansoprazole (orodispersible tablets, 30 mg daily)<br><br>Crystalloid solutions 500/1000 ml	Administered for at least 14 days given the high prevalence of reduced mobility<br><br>Administered for at least 7 days<br><br><br><br>As long as needed based on dehydration signs
Red code (moderate to severe symptoms)	Prophylactic enoxaparin (4.000 U.I. aXa/0,4 ml - dose adjustment in case of severe renal impairment)<br><br>Dexamethasone 6-16 mg daily im<br><br>Antibiotic treatment using 3rd generation cephalosporins (e.g. ceftriaxone 2 g daily) and/or macrolides (azithromycin 500 mg daily)<br><br>Lansoprazole (orodispersible tablets, 30 mg daily)<br><br>Crystalloid solutions 500/1000 ml	Administered for at least 14 days given the high prevalence of reduced mobility<br><br>Administered for at least 7 days<br><br><br><br>As long as needed based on dehydration signs

The table provides a simplified summary of medical therapy that was adopted and discussed in each patient with COVID-19 infection. Therapeutic reconciliation was always performed and deprescribing was carried out when deemed appropriate, e.g. reduction or withdrawal of psychoactive drugs in case of drowsiness or psychomotor slowing, discontinuation of antihypertensive medications in case of low blood pressure.

Table S2. Strategies for prevention and management of geriatric syndromes.

Geriatric syndrome	Prevention and management
DELIRIUM	Early identification of high risk patients; definition of patients' baseline cognitive functioning and detection of any cognitive change; clear communication; early mobilization; avoidance of mechanical restraints, limited use of device (e.g. bladder catheters) and psychoactive medications; use of non-pharmacological strategies to promote sleep and reduce anxiety; use of environmental stimuli to promote patients orientation; noise reduction strategies; provision of glasses and other aids for sensory deficits; pain control; proper nutrition and hydration; continence monitoring to avoid constipation and bladder obstruction. In case delirium occurs: non-pharmacological strategies as first line treatment approach addressing precipitating factors; pharmacological strategies as second line therapy.
HYPOKINETIC SYNDROME	Early mobilization protocols, scheduled repositioning programs, daily walking training and physical exercises. Limited use of bladder catheters and physical restraints, whenever possible.
PRESSURE SORES	Early identification of high risk patients through validated scales; routine skin inspection and hydration; early mobilization, scheduled repositioning programs (full change position at least every 2hrs), rehabilitation protocols as appropriate; use of pressure-relieving surfaces; proper nutrition and hydration, including nutritional supplements if needed; avoidance of skin moisture using barrier protection, proper continence management with pads and regular hygiene; use of sheet for bedridden patients' mobilization.
URINARY INCONTINENCE	Accurate diagnostic assessment including type of incontinence and potential causes; scheduled toileting; incontinence undergarments or pads, medical therapy review, use of medical therapy (α-agonist, a-antagonists, estrogen) and catheters in selected patients.
CONSTIPATION	Early and regular mobilization; fiber-rich diet, adequate hydration; medical therapy review to avoid/reduce potentially causative medications (e.g. opioids); monitoring of bowel function; use of laxatives and enema, when necessary.
MALNUTRITION	Estimate of daily protein and caloric intake; identification of high risk patients using validated scales (e.g. Mini Nutritional Assessment); personalized diet protocols including favorite food, caloric, protein and vitamins supplements if needed; feeding assistance; specific diets for dysphagia; social eating; enteral or parenteral nutrition in selected cases.
